# A computational model of the DNA damage-induced IKK/ NF-κB pathway reveals a critical dependence on irradiation dose and PARP-1

**DOI:** 10.1016/j.isci.2023.107917

**Published:** 2023-09-14

**Authors:** Fabian Konrath, Michael Willenbrock, Dorothea Busse, Claus Scheidereit, Jana Wolf

**Affiliations:** 1 Mathematical Modelling of Cellular Processes, Max Delbrueck Center for Molecular Medicine in the Helmholtz Association (MDC), Berlin, Germany; 2 Laboratory for Signal Transduction in Tumor Cells, Max Delbrueck Center for Molecular Medicine in the Helmholtz Association (MDC), Berlin, Germany; 3 Department of Mathematics and Computer Science, Free University Berlin, Germany

**Keywords:** Experimental models in systems biology, Systems biology

## Abstract

The activation of IKK/NF-κB by genotoxic stress is a crucial process in the DNA damage response. Due to the anti-apoptotic impact of NF-κB, it can affect cell-fate decisions upon DNA damage and therefore interfere with tumor therapy-induced cell death. Here, we developed a dynamical model describing IKK/NF-κB signaling that faithfully reproduces quantitative time course data and enables a detailed analysis of pathway regulation. The approach elucidates a pathway topology with two hubs, where the first integrates signals from two DNA damage sensors and the second forms a coherent feedforward loop. The analyses reveal a critical role of the sensor protein PARP-1 in the pathway regulation. Introducing a method for calculating the impact of changes in individual components on pathway activity in a time-resolved manner, we show how irradiation dose influences pathway activation. Our results give a mechanistic understanding relevant for the interpretation of experimental and clinical studies.

## Highlights

• A quantitative mechanistic model of DNA damage-induced IKK/NF-κB activation
• A method to quantify contributions of pathway components in signal transmission
• Regulation of signal transmission strongly depends on irradiation dose
• PARP-1 inhibition is shown to be most effective to prevent IKK/NF-κB activation

## Graphical abstract

**Figure:**
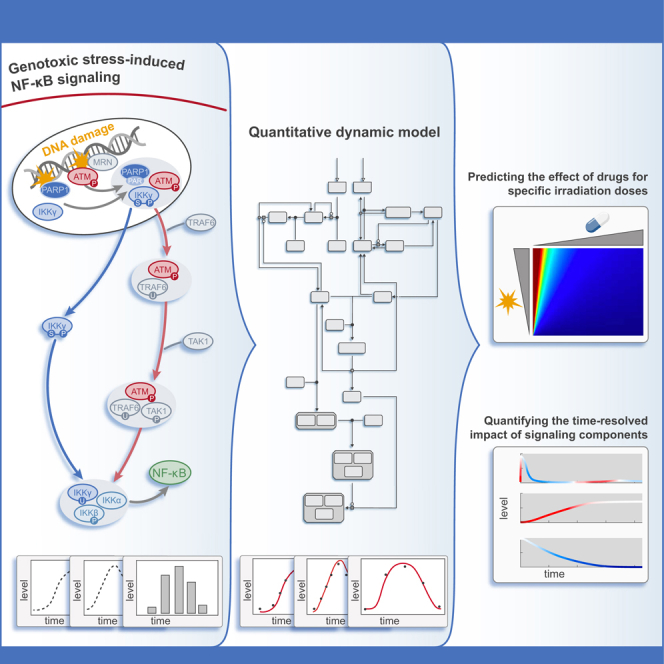

## Introduction

The members of the NF-κB transcription factor family are involved in the regulation of important cellular processes such as proliferation and survival. Over the past decades, numerous studies revealed the fundamental role of NF-κB in the regulation of inflammation, immunity as well as the cell-fate decision between survival and apoptosis. Based on the stimulus and the involved components, NF-κB signaling can be divided into three pathways: The canonical, non-canonical, and genotoxic stress-induced signaling cascade. While cell-surface receptors such as cytokine or antigen receptors trigger canonical and non-canonical signaling, genotoxic stress signals, referred to here primarily as induced by genomic DNA double-strand breaks, emanate from the nucleus.

Despite their differences, all three signaling systems culminate in the activation of NF-κB transcription factors in various crucial cellular processes, therefore underscoring the detrimental consequences of dysregulated NF-κB activity. Auto-immune diseases like psoriasis or the survival of cancer cells are examples of hyperactivated NF-κB.^1^ Hence, the biochemical interaction networks involved in the regulation of NF-κB activation were the subject of numerous studies. For the canonical pathway and parts of the non-canonical pathway, computational models were developed and their systematic and detailed analyses greatly contributed to the understanding of the regulation of the NF-κB family members.^2^ In particular, multiple ODE models of the canonical pathway were developed to study the dynamical behavior of NF-κB,^3^^,^^4^^,^^5^ its regulation via IκBs and A20^6^^,^^7^^,^^8^^,^^9^^,^^10^ as well as its role in the B cell development.^11^ For the non-canonical pathway, ODE models were used to elucidate the impact of NF-κB precursors and their processing on the regulation of NF-κB signaling.^12^^,^^13^

For the DNA damage-induced signaling pathway, seminal experimental work revealed the signaling components involved in transferring the signal emerging from DNA lesions to the activation of NF-κB and the concomitant expression of its target genes (reviewed by McCool K.W. and Miyamoto S.^14^). In general, a cell is permanently challenged by various kinds of external and internal stresses. For instance, UV irradiation or reactive oxygen species are genotoxic stresses that can damage DNA and thereby cause genomic instability.^15^^,^^16^ To prevent malignant transformation, cells need to respond to such DNA lesions in an appropriate way. The DNA damage response comprises recognition of damaged DNA, signal transduction, and the final cellular response.^17^ The processes and pathways that are involved in the signal transduction are dependent on the damage type. The most severe damage is DNA double-strand breaks (DSBs). Homologous recombination and non-homologous end-joining are two pathways that allow the repair of DSBs. However, in case of complex or irreparable damage, apoptosis or a permanent cell-cycle arrest program termed senescence is induced.^18^^,^^19^ The p65/p50 heterodimer (from here on referred to as NF-κB) is a member of the NF-κB transcription factor family and is activated during the DNA damage response.^14^^,^^20^ Similar to the canonical pathway, the activity of NF-κB is tightly controlled by IKK, a complex consisting of kinase IKKα (IKK1), kinase IKKβ (IKK2), and the regulatory subunit IKKγ (NEMO).^21^ Upon activation, IKKβ induces the activation of NF-κB by mediating the phosphorylation and proteasomal degradation of IκB proteins, inhibitors of NF-κB, that sequester NF-κB in the cytoplasm.^22^ Moreover, IKKβ mediates posttranslational modifications of p65 which are crucial for full activation of NF-κB.^21^^,^^23^ For genotoxic stress-induced IKK/NF-κB signaling, IKKγ plays a central role by transferring the signal from the nucleus to the cytoplasm.^14^

First, the sensors poly(adenosine diphosphate (ADP)-ribose) polymerase 1 (PARP-1) and the MRN complex, consisting of MRE11, RAD50, and NBS1, detect DNA double-strand breaks. The MRN complex activates the kinase ATM.^24^^,^^25^ Activated PARP-1 and ATM form a nuclear complex with IKKγ, which leads to the posttranslational modifications of IKKγ in the nucleus.^26^ The genotoxic stress-induced signal is then transferred to the cytoplasm by shuttling of modified IKKγ to the cytoplasm and its integration into IKK complexes.^14^^,^^27^^,^^28^ In parallel, phosphorylated ATM translocates to the cytoplasm and mediates the formation of a TRAF6 and TAK1-containing complex which in turn facilitates the activation of the primed IKK complexes by the phosphorylation of the IKKβ subunit.^28^ The activated IKK complex phosphorylates IκBα and thereby mediates the proteasomal degradation of the NF-κB inhibitor.^22^ Freed NF-κB can translocate into the nucleus, where it induces the expression of various target genes encoding anti-apoptotic factors such as XIAP, c-FLIP, BCL-XL, and BCL2^29^^,^^30^^,^^31^^,^^32^ that counteract apoptosis and thereby promote cell survival.^33^

Due to its impact on cell-fate decisions, aberrant functionalities of NF-κB can result in tumor development as well as tumor progression.^34^ Activated NF-κB is observed in several types of cancer^35^^,^^36^ and may mediate resistance to apoptosis, a known critical event in tumorigenesis.^16^ Importantly, the choice between cell death and survival upon DNA damage is also of great importance for tumor therapy as a common therapeutic strategy is to induce DNA damage and promote cell death of tumor cells.^37^^,^^38^ Hence, chemotherapy as well as radiotherapy trigger the activation of NF-κB,^39^^,^^40^^,^^41^ which in turn may interfere with therapy-induced cell death. Consequently, blocking NF-κB activity can enhance the susceptibility of tumor cells to therapy.^42^^,^^43^

Hence, genotoxic stress-induced IKK/NF-κB signaling is of importance to advance the understanding of tumor development and improved therapeutic strategies. To gain mechanistic insights into oncogenic signaling pathways, quantitative computational models have been shown to be powerful tools.^44^^,^^45^

Here, we developed a dynamic model of the IKK/NF-κB activation induced by DNA damage. The model, consisting of a system of coupled ordinary differential equations, is based on the biochemical network described in the experimental literature. For most of the components involved in genotoxic stress signaling, profound time-resolved data exist for different irradiation doses and cell types used in the different studies. We integrated the ample experimental observations on a quantitative level and were able to create a computational model that faithfully describes IKK/NF-κB signaling in tumor cell lines. We then used the model for a detailed analysis of the regulation of IKK/NF-κB signaling triggered by DNA damage. Introducing a new method that allows to assess the impact of changes in individual signaling components on a process in a temporally resolved and systematic manner, we could show that the regulation of the signaling is a highly dynamic process that depends on the irradiation dose. The analysis allowed the identification of potential drug targets with a major impact on inhibiting the activation of the IKK complex for different irradiation doses.

## Results

### A mathematical model describing the activation of IKK/NF-κB signaling by genotoxic stress

**Figure 1:**
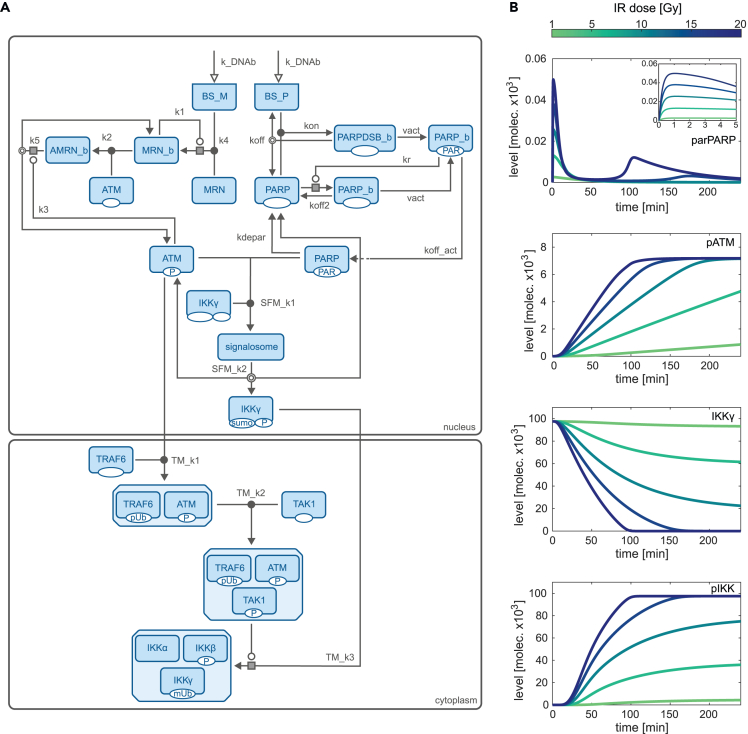
Model scheme and simulations of selected pathway components (A) The model describes the following nuclear processes: PARylation of PARP-1 upon its recruitment to the binding site BS_P, recruitment of MRN to binding site BS_M, activation of ATM and formation of the signalosome consisting of PARylated PARP (parPARP), phosphorylated ATM (pATM), and IKKγ (IKKγ). Posttranslationally modified IKKγ (spIKKγ) and pATM translocate to the cytoplasm. There, the formation of the ATM-containing TRAF6 complex (AT) and the ATM-TAK1-TRAF6 complex (ATT) as well as the activation of the IKK complex (pIKK) are taken into account. PARP-1 can either bind directly to the lesion (PARPDSB_b) or it is recruited by PARylated and DNA bound PARP-1 molecules (PAR-PARP_b) to the proximity of the lesion (PARP_b). The MRN complex is recruited to the lesion (MRN_b) and in turn recruits ATM (AMRN_b). Based on the SBGN convention,^46^ posttranslational modifications are indicated below the boxes that represent network components. P: phosphorylation, PAR: PARylation, sumo: sumoylation, pUb: poly-ubiquitination, mUb: mono-ubiquitination. White arrow heads represent inputs, white circles pointing to a gray box represent catalytic processes in which a component modulates the process with the gray box, double line circles show dissociation processes and black circles represent association processes. For a detailed description of the model processes, see STAR methods. (B) Simulation results for parPARP, pATM, IKKγ, and pIKK for an irradiation dose of 1, 5, 10, 15, and 20 Gy.

We developed an ordinary differential equation-based model based on literature-derived mechanisms and experiment-informed refinements (for details of the model development and incorporated experiments see STAR methods). The model describes the initial recruitment of the DNA damage sensors PARP-1 and the MRN complex to DNA lesions, the integration of the signal, and its transmission from the nucleus to the cytoplasm via IKKγ and ATM as well as the final activation of the IKK complex (see Figure 1A for a model scheme and STAR methods for model details). In the presence of DSBs, the recruited MRN complex mediates the recruitment and activation of the kinase ATM.^47^^,^^48^ PARP-1 undergoes automodification by attaching poly(ADP-ribose) (PAR) molecules to itself and thereby forms a platform for the activated ATM, the SUMO-1 ligase PIASy, and IKKγ.^26^ The close proximity of the components facilitates the phosphorylation and sumoylation of IKKγ.^26^^,^^49^^,^^50^ The nuclear complex, termed signalosome, disintegrates due to dePARylation of PARP-1 which is mediated by the PAR glycohydrolase.^51^ The complex disassembly frees the posttranslationally modified IKKγ and enables its translocation to the cytoplasm^14^^,^^26^^,^^27^ where it integrates into an IKK complex.^26^

**Figure 2:**
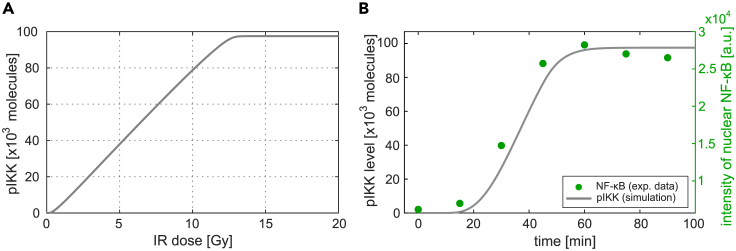
Input—output behavior of the model and comparison of simulated IKK complex activation and experimentally quantified NF-κB activation (A) For different irradiation doses (input), the corresponding stimulated steady-state level of activated IKK complex (pIKK, output) is simulated. (B) The green dots depict quantified EMSA data of nuclear NF-κB detected in HepG2 cells after 40 Gy irradiation. The gray line represents the simulated time course of IKK complex activation.

Simultaneously, activated ATM translocates to the cytoplasm where it induces the formation of a complex containing TRAF6 and TAK1, which in turn mediates the activation of the primed IKK complexes.^28^ We trained the model on various datasets capturing for multiple irradiation doses detailed time course measurements of critical pathway components, such as PARP-1, ATM, and IKKγ (STAR methods). Using those datasets with around 10,000 data points enabled us to create a model that faithfully reproduces the dynamics of genotoxic stress-induced IKK/NF-κB signaling (for a comparison of experimental data and simulated dynamics see Figure S1). In this way, the model integrates the information of these different datasets and links them based on the underlying mechanistic network of biochemical interactions. The trained model can be used to simulate and predict the time courses of all implemented network components for various irradiation doses. Figure 1B shows the dynamics of selected pathway components: posttranslationally modified PARP-1 (parPARP), phosphorylated ATM (pATM), unmodified IKKγ (IKKγ) in the nucleus, and activated cytoplasmic IKK complex (pIKK) consisting of IKKα, phosphorylated IKKβ, und posttranslationally modified IKKγ. The simulations reveal different activation dynamics of the two DNA damage sensors. While PARP-1 is fast and transiently modified (parPARP) with a peak amplitude depending on the irradiation dose, the levels of pATM increase until a constant level is reached. Note that the model component parPARP represents a subset of the pool of PARylated PARP-1 molecules which is modified and dissociated from DNA. The slope of the pATM increase strongly depends on the irradiation dose with a faster increase for higher irradiations. Unmodified IKKγ (IKKγ) is declining over time and the levels of activated IKK complex (pIKK) increase over time. For both components, the slope and activated steady-state levels change with irradiation levels. A full activation of the IKK complex is reached for around 13 Gy and higher (Figure 2A).

To validate the model, we compared the simulated time course of the activated IKK complex and measured levels of nuclear NF-κB upon 40 Gy γ-irradiation, a dataset that was not used for parameter estimation. We find a good agreement between simulation and this experimental dataset (Figure 2B), which demonstrates that (i) the level of activated IKK complex is a good approximation for NF-κB activity and (ii) the model is able to successfully reflect the dynamics of NF-κB activity upon γ-irradiation. We therefore define for the following analyses activated IKK complex as readout (see STAR methods for details).

### Model predicts PARP-1 inhibition as most effective target to reduce IKK complex activity

**Figure 3:**
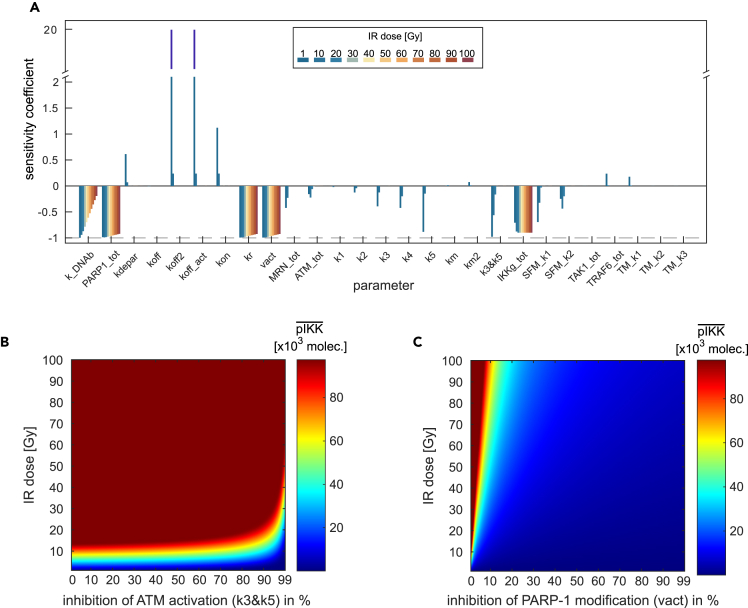
Impact of parameter perturbations on the level of activated IKK complex for various irradiation doses (A) The values of the specified parameters were reduced by 90% for various irradiation doses. The effect of a parameter perturbation on the stimulated steady state level of the activated IKK complex is given by the sensitivity coefficient. Minus one (dashed line) indicates the maximal inhibition of IKK complex activation resulting in an activated IKK complex level of zero after parameter perturbation. Parameters with the suffix *tot* represent the total amount of a conserved moiety. To quantify the effect of ATM inhibition, an additional sensitivity coefficient was calculated for the two parameters representing ATM activation (k3 and k5, denoted by k3&k5). Both parameters were simultaneously reduced by 90%. (B and C) ATM activation and PARylation of PARP-1, respectively, are inhibited for various efficiencies ranging from 0% inhibition (unperturbed) to 99% inhibition. The color in the plots indicate the amount of activated IKK complex (pIKK) molecules in the stimulated steady state. The inhibition of ATM activation is simulated by a simultaneous perturbation of the two parameters k3 and k5, the inhibition of PARylation of PARP-1 by perturbation of the parameter vact.

Due to the anti-apoptotic activity of NF-κB, it can be beneficial to inhibit its activation upon tumor therapy and thereby render tumor cells sensitive to therapy-induced apoptosis.^36^ Thus, we sought to identify processes that would allow us to effectively interfere with the activation of IKK/NF-κB. We therefore performed a sensitivity analysis on all model parameters for a broad range of irradiation doses ranging from 1 to 100 Gy. The parameters were decreased by 90% with respect to their nominal value, and the change in the stimulated steady state of activated IKK complex (pIKK) was quantified (Figure 3A).

The parameters with the strongest negative impact on IKK activity for all tested irradiation doses are the total amount of PARP-1 (PARP1_tot) and the rate constants of PARP-1 recruitment (kr) and PARP-1 auto-modification (vact). The total amount of IKKγ (IKKg_tot) also has a negative impact on IKK complex activity for all tested irradiation doses but to a lower extent than the parameters affecting PARP-1 modification. The dissociation rate parameters koff2 and koff_act (characterizing the dissociation of PARP-1 from DNA) have a strong positive effect on the level of activated IKK complex as a reduction of those parameters causes an increase in the amount of modified PARP-1 and a stronger IKK complex activation. Interestingly, for the parameters koff2, koff_act, but also others, the sensitivity coefficients strongly depend on the irradiation dose. This is also true for an inhibition of ATM activation, represented in Figure 3A by parameter label k3&k5, which strongly reduces the level of activated IKK complex for 1 Gy but has only a minor effect for higher irradiation doses (Figure 3A). To analyze the dependency of the sensitivities on the applied irradiation dose in more detail, we focused on the effect of two processes, namely activation of ATM (k3&k5) and PARylation of PARP-1 (vact), which are targets of clinically relevant inhibitors.^52^^,^^53^ We simulated the level of activated IKK complex in the stimulated steady state for different inhibition efficiencies of ATM activation and PARP-1 modification as well as for various irradiation doses (Figures 3B and 3C). With unperturbed ATM activity (inhibition efficiency: 0%), the amount of activated IKK complex increases with increasing irradiation doses until the maximal level of activated IKK is reached (Figure 3B). Inhibition of ATM activity by 90% reduces the level of activated IKK, however, this is only valid for irradiation doses below 25 Gy. For higher irradiation doses, a 90% inhibition of ATM activity is not sufficient to decrease the level of activated IKK complex. In order to reduce the level of activated IKK complex in the range of high-irradiation doses, the inhibition efficiency of ATM activity needs to be increased. For example, for an irradiation dose of around 80 Gy even a 99% inhibition of ATM activity has only a negligible effect on the IKK complex activity. In contrast, an inhibition of PARP-1 auto-modification has for all irradiation doses a much stronger effect on the level of activated IKK complex (Figure 3C). Even for an irradiation dose of 100 Gy, inhibition of PARP-1 auto-modification by 50% is sufficient to strongly reduce the amount of activated IKK complex. Consequently, these results reveal that activation of IKK complex and thus the activity of NF-κB is more sensitive to PARP-1 inhibition than to ATM inhibition.

### Elucidating the impact of changes in pathway components uncovers an irradiation dose-dependent shift in critical components

To mechanistically understand the results of the sensitivity and inhibitor analysis, we investigated the regulation of the pathway activation and its dependence on the irradiation dose in more detail. The derived pathway model for IKK/NF-κB activation exhibits two hubs at which two signaling branches converge, respectively (see Figure 1). The first hub is the formation of the signalosome which connects the branches of PARP-1 modification and ATM phosphorylation. At the second hub, the IKK complex is activated by posttranslationally modified IKKγ (spIKKγ) and pATM-mediated complex formation in the cytoplasm.

We here focus on the first hub, the formation of the signalosome. Within the signalosome, IKKγ becomes posttranslationally modified, which makes that hub a critical step in the transduction of the signals. In order to understand the regulation of the signalosome formation in a time-resolved manner, we analyzed the impact of the changes in each of the three components, unmodified IKKγ, parPARP, and pATM, on the flux of signalosome formation (Equation 1). We, therefore, developed a new method that allows determining the impact of changes in components on a given flux by calculating the normalized derivative of flux for each time point (Equation 2). This way, the change in the flux is composed of the normalized change of each component and allows to assign the impact of individual changes in the components to the change in the flux at a given time point. To quantify this impact, we defined the time-resolved impact fraction *ω* for each component (Equations 3, 4, and 5).

(Equation 1) v(t)=k·x(t)·y(t)·z(t)

withk≔SFM_k1,x≔parPARP,y≔pATM,z≔IKKγ

(Equation 2) $$\frac{dv}{dt}\cdot \frac{1}{v}=\frac{dx}{dt}\cdot \frac{1}{x}+\frac{dy}{dt}\cdot \frac{1}{y}+\frac{dz}{dt}\cdot \frac{1}{z}$$

(Equation 3) $$\omega_{x}=\frac{\frac{dx}{dt}\cdot \frac{1}{x}}{\left|\frac{dx}{dt}\right|\cdot \frac{1}{x}+\left|\frac{dy}{dt}\right|\cdot \frac{1}{y}+\left|\frac{dz}{dt}\right|\cdot \frac{1}{z}}$$

(Equation 4) $$\omega_{y}=\frac{\frac{dy}{dt}\cdot \frac{1}{y}}{\left|\frac{dx}{dt}\right|\cdot \frac{1}{x}+\left|\frac{dy}{dt}\right|\cdot \frac{1}{y}+\left|\frac{dz}{dt}\right|\cdot \frac{1}{z}}$$

(Equation 5) $$\omega_{z}=\frac{\frac{dz}{dt}\cdot \frac{1}{z}}{\left|\frac{dx}{dt}\right|\cdot \frac{1}{x}+\left|\frac{dy}{dt}\right|\cdot \frac{1}{y}+\left|\frac{dz}{dt}\right|\cdot \frac{1}{z}}$$

Note that the absolute values of the impact fractions of the three components sum up to one, thereby allowing to compare their impact on the derivative of flux *v*.

**Figure 4:**
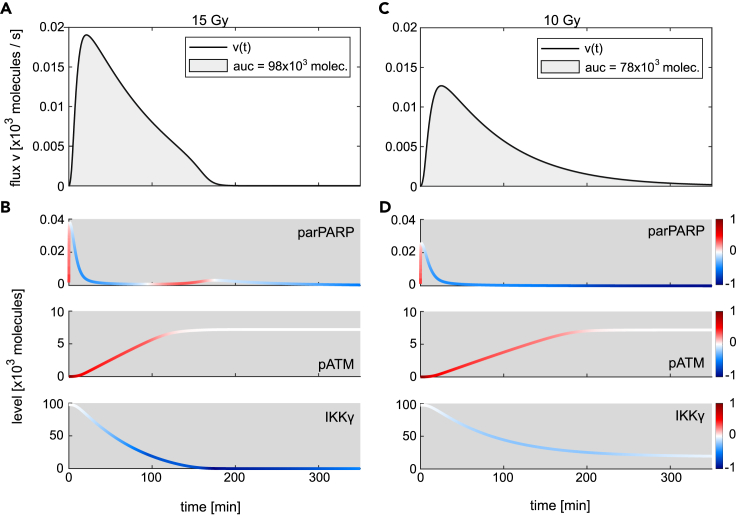
Influence of changes in parPARP, pATM, and IKKγ on the signalosome formation flux (A) The black line shows the simulated time course of the flux of signalosome formation for 15 Gy irradiation. The area under the curve (auc) of the flux (filled in gray) corresponds to the total number of modified IKKγ molecules. (B) The trajectories of PARylated PARP-1 (parPARP), phosphorylated ATM (pATM), and IKKγ are colored based on the impact fraction *ω* of the respective component at a given time point. The value of an impact fraction can range from plus one and minus one. While red colored parts of a trajectory represent a positive value and a positive impact on the flux, blue represents a negative value and a negative impact, white shows a negligible impact. (C and D) Simulated time course of signalosome formation upon 10 Gy and the corresponding trajectories of parPARP, pATM, and IKKγ.

We first analyzed the signalosome formation for an irradiation dose of 15 Gy which leads to a full activation of the IKK complex (Figure 2A). Figure 4A shows the signalosome formation flux over time; Figure 4B shows the dynamics of PARylated PARP-1, phosphorylated ATM, and IKKγ. The impact of the change in each component at a given time point is calculated according to Equations 3, 4, and 5, and the value for fraction *ω* is color coded for each component at the corresponding time point and overlayed with its time course (Figure 4B). Comparing the trajectories in Figure 4B with the flux of signalosome formation (Figure 4A) reveals that the increase in the flux of signalosome formation within the first minutes is caused by both, the increase in the level of parPARP and pATM, which is visualized by the red color of the corresponding trajectories. After the initial increase of parPARP, it rapidly decreases and negatively affects the flux of signalosome formation, indicated by the blue color. However, the flux of signalosome formation further increases during the first 20 min since the levels of pATM positively affect the flux while the decrease in IKKγ levels does not affect it given by the white color of the trajectory of IKKγ. Thus, after the initial increase of parPARP, pATM is the only component positively affecting the flux during this time period and is therefore solely responsible for the flux to further increase. After around 20 min, the flux of signalosome formation starts to decrease and eventually becomes zero (at around 190 min). Within this time frame, the change in parPARP has initially a negative impact on the flux and based on the darker blue color compared to that of the change in IKKγ, it drives in the beginning the decrease of the flux. However, at around 100 min, the levels of parPARP show another, but minor increase resulting in a positive impact on the flux that counteracts, together with the positive impact of the change in pATM, the negative impact of the change in IKKγ. Between 20 and 190 min, the negative influence of IKKγ becomes stronger and ultimately IKKγ becomes the critical component determining the decrease of the flux which finally leads to the abrogation of signalosome formation.

In order to analyze the regulation of the pIKK complex level for irradiation doses that do not lead to maximal activation of the system, we computed the time-resolved impact fractions for the signalosome formation for 10 Gy (Figures 4C and 4D). For this lower irradiation dose, the flux of signalosome formation is lower and while the flux increase has a similar timing, the decrease of the flux takes much longer. Comparing the dynamics of parPARP, pATM, and IKKγ reveals slight changes in concentrations and timings. Importantly, the impact fractions of parPARP and IKKγ show strong changes between the two irradiation doses. In contrast to the 15 Gy irradiation, the level of IKKγ does not affect signalosome formation which is indicated by a time-resolved impact fraction close to zero throughout the whole time frame of 350 min (IKKγ, Figure 4D). Similar to the 15 Gy irradiation scenario, the initial increase in the parPARP level positively contributes to the increase in the signalosome formation together with the increase in the pATM level. While parPARP decreases, pATM further increases and drives the increase in the flux. However, the impact of decreasing parPARP levels on the flux rises and becomes close to minus one which finally leads to the abrogation of signalosome formation.

Overall, the analyses demonstrate that the initial signalosome formation is positively impacted by changes in parPARP and pATM levels, enabling integration of signals via the two branches of DNA damage sensor signaling. The abrogation of signalosome formation is controlled by parPARP and IKKγ. Here, the main control differs for different irradiation doses. While for 15 Gy and above (leading to full IKK complex activation) IKKγ essentially prevents further signalosome formation, for 10 Gy parPARP has the main control on signalosome formation.

Elucidating the control properties of signalosome formation is important as it determines the amount of IKKγ that can be posttranslationally modified during the signaling response. From the model structure, one can derive that the amount of modified IKKγ (spIKKγ) determines the level of activated IKK complex in the stimulated steady state (STAR methods). This can be demonstrated by integrating the flux of signalosome formation, which corresponds to the total amount of modified IKKγ in the nucleus over time (gray areas under the curves, Figures 4A and 4C). The calculated molecule numbers for 15 Gy and 10 Gy equal the level of activated IKK complex in a steady state for the corresponding irradiation doses (Figure 2A). Consequently, the irradiation dose affects the regulation of IKK activity by causing a switch in critical components that restrict signalosome formation.

### ATM inhibition modulates the availability of PARylated PARP-1 for signalosome formation

The results of the sensitivity and inhibitor analysis (Figure 3) revealed that for higher irradiation doses the level of activated IKK complex in the stimulated steady state is more sensitive to inhibition of PARP-1-related processes of PARylation (e.g., parameter vact) than ATM inhibition (parameter k3&k5). To understand the weaker effect of ATM inhibition on the level of activated IKK complex, we evaluated the effect of this inhibition on the time-resolved impact fractions of parPARP, pATM, and IKKγ during signalosome formation.

**Figure 5:**
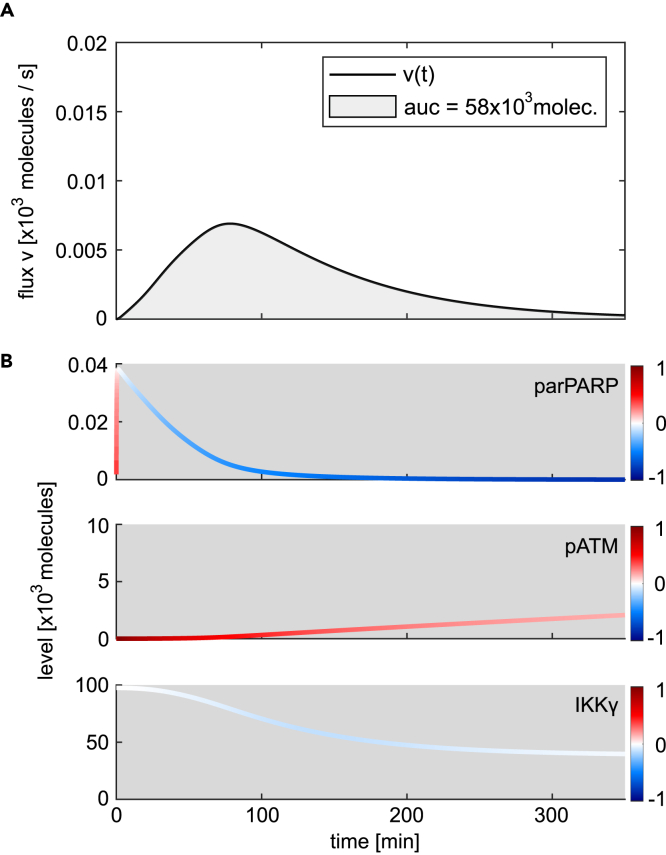
Change in the time-resolved impact fractions of parPARP, pATM, and IKKγ at 15 Gy and ATM inhibition (A) Simulated time course of the flux of signalosome formation and area under the curve (auc) upon 15 Gy and inhibition of ATM activation by 90%. The inhibition is simulated by a simultaneous decrease in parameters k3 and k5 by 90%. (B) Trajectories of parPARP, pATM, and IKKγ are colored based on the corresponding time-resolved impact fraction ω.

Figure 5A and 5B show the dynamics of the signalosome formation flux and the trajectories of parPARP, pATM, and unmodified IKKγ, respectively, for an irradiation dose of 15 Gy and a 90% inhibition of ATM activation. As expected from the inhibitor analysis shown in Figure 3B, a 90% ATM inhibition at an irradiation dose of 15 Gy causes a reduction of the level of activated IKK complex in the cytoplasm. This reduced amount of activated IKK complex is reflected by a smaller area under the curve of the flux of signalosome formation (compare Figure 4A, 98,000 species; Figure 5A, 58,000 species).

The time course in Figure 5B demonstrates that inhibition of ATM activity results in strongly reduced pATM levels during the initial increase of the flux of signalosome formation. Consequently, the increase in the flux is less steep, the maximal value is reduced, and the time frame of signalosome formation is prolonged (compare Figures 5A and 4A). Strikingly, with ATM inhibition, after 80 min, the reduction in the level of PARylated PARP-1 becomes critical and drives the decrease of the flux of signalosome formation until the flux is abrogated (Figure 5). The decrease in the level of unmodified IKKγ also contributes to the decrease in the flux (light blue color of the IKKγ trajectory in Figure 5B) but to a much smaller extent than the change in the parPARP level (dark blue color of the parPARP trajectory in Figure 5B). The level of phosphorylated ATM increases during signalosome formation and has a positive impact on the flux (red color of the pATM trajectory). However, the positive influence is counteracted by the strong negative impact of decreasing parPARP levels. Hence, inhibition of ATM activation reduces the amount of available parPARP for signalosome formation which establishes a regulatory regime, similar to the signalosome formation for an irradiation dose of 10 Gy (Figure 4D) where parPARP has the main limiting role in signalosome formation.

Based on these results, one can understand the different efficacies of ATM and PARP-1 inhibition with respect to reducing the level of activated IKK complex as it was shown in Figure 3. As can be seen in Figures 3A and 3B, ATM inhibition controls the level of activated IKK complex only up to a certain irradiation dose. This effect is mediated by a reduction of the availability of parPARP for signalosome formation (Figure 5). However, for higher irradiation doses, the amount of parPARP is increased (Figure 1B) and thereby counteracts the effect of ATM inhibition. To quantify the overall amount of parPARP that is produced and therefore available during signalosome formation, we calculated its cumulated level in the corresponding time frame (Figure S2, gray line). As expected, the cumulated amount of available parPARP increases with increasing IR doses. In contrast, inhibition of PARP-1 PARylation strongly reduces the amount of parPARP for all indicated irradiation doses (Figure S2, orange line).

In summary, inhibition of ATM activation can reduce the level of activated IKK complex in the stimulated steady state by limiting the availability of PARylated PARP-1 and thereby reducing the flux of signalosome formation and consequently the amount of modified IKKγ. As increasing irradiation doses lead to higher levels of PARylated PARP-1, ATM inhibition is only effective in a certain range of irradiation doses.

### Parallel signaling branches from the nucleus to cytoplasm shape the dynamics of IKK complex activation

We so far analyzed the signal integration in the first hub of the pathway that is the signalosome formation in the nucleus. We now focus on the second hub, the activation of the IKK complex in the cytoplasm. Evaluating the time-resolved regulation of the second hub is of interest as it sheds light on the initiation of IKK complex activity and therefore the onset of NF-κB activity upon generation of DSBs. To assess the regulation of initial IKK complex activation, we calculated the time-resolved impact fractions of spIKKγ and the cytoplasmic TRAF6 complex (ATT) for the flux of IKK complex activation (Equation 6). We chose these two components as their changes have a direct impact on the activation of the IKK complex. Based on the normalized derivative of the flux (Equation 7) we calculated the impact fractions for spIKKγ, *ω*_*x*_ (Equation 8), and ATT, *ω*_*y*_ (Equation 9).

(Equation 6) v(t)=k·x(t)·y(t)

withk≔TM_k3,x≔spIKKγ,y≔ATT

(Equation 7) $$\frac{dv}{dt}\cdot \frac{1}{v}=\frac{dx}{dt}\cdot \frac{1}{x}+\frac{dy}{dt}\cdot \frac{1}{y}$$

(Equation 8) $$\omega_{x}=\frac{\frac{dx}{dt}\cdot \frac{1}{x}}{\left|\frac{dx}{dt}\right|\cdot \frac{1}{x}+\left|\frac{dy}{dt}\right|\cdot \frac{1}{y}}$$

(Equation 9) $$\omega_{y}=\frac{\frac{dy}{dt}\cdot \frac{1}{y}}{\left|\frac{dx}{dt}\right|\cdot \frac{1}{x}+\left|\frac{dy}{dt}\right|\cdot \frac{1}{y}}$$

**Figure 6:**
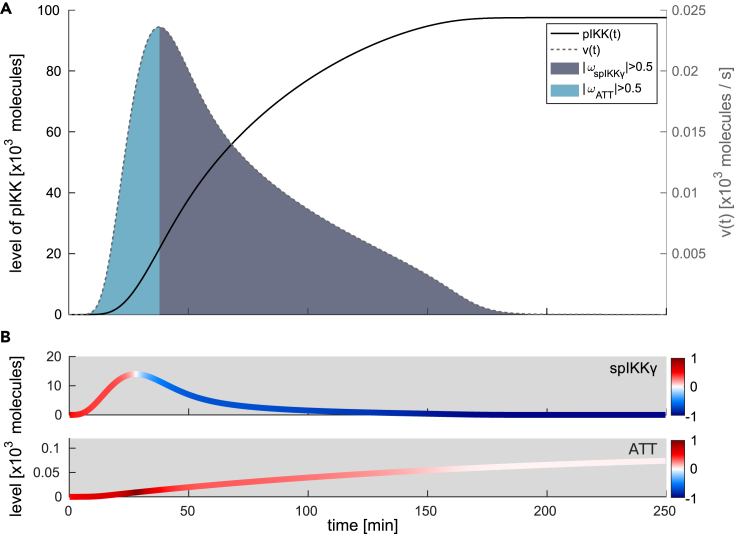
Influence of changes in sumoylated and phosphorylated IKKγ and the ATM-TRAF6-TAK1 complex on IKK complex activation upon 15 Gy (A) The black solid line represents the simulated level of activated IKK complex (pIKK) over time. The flux of IKK complex activation is shown as gray dashed line. The area under the curve has a color-code according to the component change exerting the dominant impact on IKK complex activation at a given time point. The gray color corresponds to a predominant impact of changes in the sumoylated and phosphorylated IKK (spIKKγ) and blue to a predominant impact of changes in the ATM-TRAF6-TAK1 (ATT) complex. (B) Level of spIKKγ and ATT over time. The trajectories are colored based on the impact fraction of the respective component at a certain time point. A red color represents a positive impact on the flux of IKK complex activation and blue represents a negative impact.

Figure 6A shows the level of activated IKK complex (pIKK, solid line) as well as the flux of IKK complex activation (dashed line) upon 15 Gy irradiation. After around 10 min, the level of pIKK starts to increase until it reaches the stimulated steady-state level after around 200 min (Figure 6A, solid line). Figure 6B depicts the dynamics of spIKKγ and ATT as well as their color-coded impact fractions. The impact fractions allow us to identify the critical component driving the changes in the flux of IKK complex activation. We defined a threshold of 50% for the absolute value of an impact fraction to determine which of the two components has the main impact on changes in the flux of IKK complex activation. We colored the time frames in Figure 6A as areas under the flux accordingly. Notably, the initial increase of the flux (Figure 6A, dashed line) is positively impacted by the increase of ATT and in parts by that of spIKKγ. ATT is the critical component during this flux increase due to a higher impact fraction compared to spIKKγ (Figure 6A, blue area). After the critical component switches from ATT to spIKKγ which is indicated by the gray area in Figure 6A, the flux does not increase further but starts to decrease. Since the decreasing levels of spIKKγ have a negative impact on the flux (blue-colored trajectory of spIKKγ), the dominating impact of changes in spIKKγ counteracts the positive impact of changes in ATT and thereby causes a decrease in the flux of IKK activation. Consequently, the level of activated IKK complex (Figure 6A, solid line) shows a reduced increase and reaches a steady state when the flux of IKK activation is zero. Hence, the initial activation of IKK complex is mediated by both components, ATT and spIKKγ, but with a main positive control of the ATM-TRAF6-TAK1-complex (ATT). The modified form of IKKγ (spIKKγ) exerts a stronger control on the level of pIKK at later time points, preventing further activation and allowing it to reach a pIKK steady state.

## Discussion

Genotoxic stress-induced IKK/NF-κB signaling plays an important role in cell fate decisions upon DNA damage and can therefore contribute to tumor development or interfere with DNA-damaging tumor therapies. To gain a systematic understanding of regulatory mechanisms within the genotoxic stress-induced IKK/NF-κB signaling network, we integrated and linked multiple datasets capturing different parts of the network from four studies^26^^,^^28^^,^^54^^,^^55^ by fitting our model to these experimental observations. This way, we developed a quantitative dynamic model describing the activation of IKK/NF-κB by genotoxic stress. Noteworthy, additional studies exist that were not used for model development but show for parts of the activation process the involvement of additional or alternative components or interactions. For example, our model implementation of IKKγ translocation is based on the study by Hinz et al. (2010) who showed that posttranslationally modified IKKγ and phosphorylated ATM translocate independently to the cytoplasm.^28^ In contrast, Jin et al.,^56^ and Wu et al.,^49^ concluded that they translocate together to the cytoplasm. Moreover, Hinz et al.,^28^ showed that auto-ubiquitination of TRAF6 leads to the formation of a complex containing IKK complexes and TAK1, while Wu et al.^57^ and Yang et al.^58^ reported that proteins such as RIPK1 facilitate the interaction between TAK1 and IKK complexes. Furthermore, the linear ubiquitination complex LUBAC has been implicated in the activation of TAK1.^59^

Despite the differences and additions, all studies report consistently that (i) IKKγ is sumoylated and subsequently ubiquitinated and (ii) TAK1 is activated by ATM and forms a poly-ubiquitin-based platform which promotes the activation of IKKβ and thereby NF-κB (for a detailed review, refer McCool K.W. and Miyamoto S.^14^). Of note, with our recent screens for components and specific inhibitors of DNA damage-induced NF-κB activation, we identified regulators as well as drug targets and re-confirmed the components used in the model, including a crucial role for PARylation.^60^^,^^61^ Our model can therefore be seen as a core model of genotoxic stress-induced IKK/NF-κB activation.

The computational analyses of the model enabled us to study the sensitivity of the pathway in detail. This revealed PARP-1 PARylation inhibition as the most sensitive targeting strategy to diminish the level of activated IKK complex for all considered irradiation doses. The prediction is in line with experimental data in which inhibition of PARP-1 PARylation blocks the genotoxic stress-induced activation of NF-κB for high-irradiation doses (Figures S1D and S1G in Stilmann et al., 2009^26^). For other targets, such as phosphorylation of ATM, the model predicts that the inhibitory effect on IKK complex activity strongly depends on the irradiation dose. Hence, the irradiation dose and therefore the number of DNA lesions determine the sensitivity of the IKK/NF-κB signaling pathway. Our results strongly support the notion that considering the irradiation dose is important to understand the regulation of IKK/NF-κB signaling by DNA double-strand breaks.^14^^,^^62^ As the applied irradiation doses can vary greatly between experimental studies (STAR methods, training and validation data sets) and between clinical studies,^38^^,^^63^ considering the irradiation dose is crucial for the evaluation and comparison of those studies.

To assess the impact of irradiation doses on the regulation of IKK complex activity in a temporal and mechanistic manner, we developed a method that allows to quantify the impact of changes in individual pathway components on a given flux. In general, this method can be applied to any ordinary differential equation-based model. Here, the method enabled us to investigate the impact of changes in three pathway components, PARP-1, ATM, and IKKγ, on the flux of signalosome formation and concomitantly the amount of activated IKK complex in the stimulated steady state. The results uncovered the availability of PARylated PARP-1 as a critical factor for signalosome formation. Since the level of phosphorylated ATM contributes to the flux of signalosome formation, it affects the amount of PARylated PARP-1 incorporated into the signalosome. Hence, our results indicate that the balance between phosphorylated ATM and PARylated PARP-1 is crucial for the regulation of IKK complex activity by genotoxic stress. It is therefore important to validate the quantities estimated for the model by parameter estimation (Table S1) with independent experimental measurements. In the study by Bakkenist and Kastan, it was shown that irradiating cells with 0.5 Gy is already sufficient to induce full activation of ATM.^48^ This is in line with our model, in which ATM is fully activated for all considered irradiation doses starting from 1 Gy. For the total amount of ATM, a value of 7 ×10^3^ molecules was estimated by model fitting which is in the same range as published values of quantified ATM molecules per cell.^64^^,^^65^ The level of total PARP-1 was set in the model to a fixed value of 1.9·10^5^ molecules based on the study of Schwanhäusser et al.,^64^ which coincides with the PARP-1 molecules per cell quantified by Beck et al.^65^ Besides ATM and PARP-1, IKKγ is also a part of the signalosome and was identified in our study as the component that limits the amount of activated IKK complex at high doses of irradiation (Figures 4A and 4B). In experiments, active NF-κB, which is approximated in our model by activated IKK (Figure 2B), can be detected upon 1 Gy irradiation and was shown to saturate in two breast cancer cell lines at 20 Gy and 50 Gy, respectively.^66^ Moreover, the number of IKKγ molecules per cell was quantified in five tested cell lines to range from 4 ×10^5^ to 2× 10^6^ molecules^67^ which is only slightly higher than the inferred 9.8× 10^4^ IKKγ molecules in our model. In conclusion, available *in vitro* studies support the predictions of our model regarding molecule numbers and proportions between ATM, PARP-1, and IKKγ as well as the input-output behavior of ATM activation and NF-κB activation. In the future, additional quantitative data on the amount of posttranslationally modified proteins could help to validate the predicted amounts and assess the impact of molecule numbers and proportions between pathway components on IKK/NF-κB signaling. Particularly, for the levels of PARylated PARP-1 the model predicts that a small amount of modified PARP-1 is sufficient to activate the IKK complex. This is due to a fast turnover of PARylated PARP-1, i.e., PARP-1 is rapidly recruited, modified, and dePARylated. Since dePARylated PARP-1 can again be recruited and modified, the calculated cumulative amount of PARylated PARP-1 is high, even for low-IR doses (Figure S2, gray line). This is in line with *in vitro* quantifications revealing fast PARylation rates,^68^^,^^69^ a short half-life of PARylated PARP-1^70^ and rapid recruitment dynamics of PARP-1 to DNA lesions (Figure S1; ^26^^,^^54^^,^^60^). Moreover, time-lapse microscopy and FRAP (fluorescence recovery after photo bleaching) experiments show that (i) only a small subset of PARP-1 molecules is recruited to the damage site,^26^^,^^54^^,^^60^ and (ii) the dissociation and subsequent recruitment of additional PARP-1 proteins at the damage site is very fast.^54^ While these observations strongly support the model prediction, a detailed quantification and analysis of the individual components of the PARylated PARP-1 pool would be necessary to quantitatively validate the fast and cyclic process of PARP-1 modification.

In addition, it would be interesting to further analyze the impact of different molecule numbers and proportions and thereby assess differences in the response of IKK/NF-κB to genotoxic stress at the level of cell types and individual cells. The current model could be used to capture such heterogeneity between cell lines and individual cells by introducing variability in parameter values based on the corresponding inferred population parameters.

Besides analzsing the regulation of signalosome formation, which is the first signaling hub in the modeled network and was predicted to control the level of activated IKK complex in a steady state, we also assessed the regulation of the second signaling hub. The parallel signaling merging in the second hub was predicted to control the initiation of activation of the IKK complex. Of note, the signal integration in the second hub forms a network motif termed coherent feedforward loop type 1, which has been shown to be able to delay the activation of the target component and filter transient inputs.^71^ By quantifying the impact of the change in posttranslationally modified IKKγ (spIKKγ) and in ATM-TRAF6-TAK1 complex (ATT) on the readout IKK complex, we could demonstrate that ATT dominantly controls the onset of IKK complex activation (Figure 6). Thus, ATT determines the delay of IKK complex activation. ATM induces the formation of the TRAF6 complex and is also crucial for the activation of the transcription factor and tumor suppressor p53 which plays a fundamental role in the genotoxic stress response. As p53 induces the expression of Wip1, a phosphatase dephosphorylating and thereby inactivating ATM, the activity of p53 can modulate the activity of ATM^72^ and therefore might be important at later time points in controlling the temporal response of NF-κB. For the second described characteristic of the feedforward motif, the filter for transient inputs, one can hypothesize that it prevents premature activation of IKK/NF-κB signaling upon minor DNA lesions. Poltz and Naumann^73^ identified multiple coherent feedforward loops in a logical model of the DNA damage response. However, the majority of the identified feedforward loops were found for p53 signaling. The importance of this network motif for the regulation of p53 was demonstrated by Loewer et al., who showed that the filter prevents p53 activation in case of transient DNA damage.^74^

Taken together, we here combined mechanistic insights and quantitative data of several experimental studies to develop a model for IKK/NF-κB activation by genotoxic stress. The analysis of the model shows how the recognition of DNA double-strand breaks by the two sensors PARP-1 and the MRN complex are integrated and transduced to the cytoplasm where the IKK complex and subsequently NF-κB is activated. Interestingly, our study reveals that the regulation properties of the pathway strongly depend on the irradiation dose. The pronounced impact of PARP-1 inhibition on IKK/NF-κB activation indicates that the anti-apoptotic impact of NF-κB might affect the outcome of clinically applied PARP-1 inhibitors. This notion is in line with experimental observations reporting PARP-1 inhibition to cause a reduction in the viability of cells in an NF-κB-dependent manner.^26^^,^^60^^,^^75^ Therefore, a consideration of the activation of IKK/NF-κB signaling by genotoxic stress could improve predicting the efficacy of PARP-1 inhibition and expand the application of inhibitors to tumor cells in which IKK/NF-κB activity is upregulated or constitutively active. The identified coherent feedforward loop is another level of regulation besides signalosome formation and might be a possible point of cross-talk between p53 signaling and IKK/NF-κB activity. Our model could be extended by interactions with the p53-signaling pathway and thereby serve as a starting point to gain a deeper understanding of cell-fate decisions imposed by genotoxic stress. It will also be an important extension for disease models capturing the cellular response to oncogenic perturbations.^76^^,^^77^

### Limitations of the study

Our study focusses on the core processes of genotoxic stress-induced IKK/NF-κB signaling. Additional components and pathways that are part of the DNA damage response, such as p53 signaling, are not included in the model but could influence the signal transduction in the modeled network. This includes processes that become relevant at later time points of genotoxic stress-induced signaling. Currently, our model covers the initial processes of IKK/NF-κB activation. However, at later time points inactivation processes like negative feedbacks regulating NF-κB activity and repair processes of the damaged DNA become a crucial part of the signaling cascade. Thus, our model represents a core model of the initial IKK/NF-κB activation that can be extended by additional components and processes to capture the genotoxic stress response in a more comprehensive way.

Moreover, our model training is based on multiple experimental datasets from different cell lines. While the model is able to reproduce and integrate all those experimental observations, cell line-specific datasets covering all parts of the signaling cascade would be necessary to confirm consistent signaling responses between cell lines. The available experimental data showing consistent recruitment dynamics of PARP-1 to DNA lesions between three cell lines support the notion of a unified response.^26^^,^^54^^,^^60^

## STAR★Methods

### Key resources table

**Table:** 

REAGENT or RESOURCE	SOURCE	IDENTIFIER
**Experimental models: Cell lines**		

HepG2	DSMZ - German Collection of Microorganisms and Cell Cultures	ACC180

**Oligonucleotides**		

H2KκB-us: 5‘-GATCCAGGGCTGGGGATTCCCCATC TCCACAGG-3‘	TIB Biomol	N/A
H2KκB-ls: 5‘-GATCCCTGTGGAGATGGGGAATCCC CAGCCCTG-3‘	TIB Biomol	N/A

**Software and algorithms**		

ImageJ	Schneider et al.^78^	https://imagej.nih.gov/ij/
Data2Dynamics modeling environment	Raue et al.^79^^,^^80^	https://github.com/Data2Dynamics/d2d
MATLAB	The Mathworks	R2017b
Mathematical model	this study	BioModels: MODEL2307130001 (https://www.ebi.ac.uk/biomodels/MODEL2307130001)

### Resource availability

#### Lead contact

Further information and requests for resources and reagents should be directed to and will be fulfilled by the lead contact, Jana Wolf (Jana.Wolf@mdc-berlin.de).

#### Materials availability

This study did not generate new unique reagents.

### Experimental model and study participant details

#### Cell culture

HepG2 cells were cultured in RPMI medium (Gibco), supplemented with 10% fetal calf serum and penicillin/streptomycin (100 U/ml and 100 μg/mL) in 95% relative humidity and 5% CO_2_.

### Method details

#### Experimental setup

##### Electrophoretic mobility shift assay (EMSA)

EMSA was performed using standard conditions^26^^,^^82^ using 3–5 μg lysate (added last) in a total volume of 20 μL containing 4% Ficoll, 20 mM HEPES pH 7.9, 60 mM KCl, 1.5 μg poly(dI-dC), 2 mM DTT, 0.1 mg/mL BSA and ca. 7 fmoles 32P-labeled H2K double stranded NF-κB binding site probe. After incubation for 30 min at 37°C, reactions were loaded to 4% (60:1 crosslinked) polyacrylamide gels in 25 mM Tris, 25 mM Borate, 0.5 mM EDTA and electrophoresed with 26 mA current for 2 h at 4°C, followed by autoradiography.

##### DNA damage induction

Cells were irradiated with a ^137^Cs source (OB29 Irradiator, STS Braunschweig) at the dose indicated.

#### Training and validation datasets

For the calibration and validation of the developed model, we obtained available datasets covering various experimental assays and irradiation (IR) doses inducing DNA damage. The following table gives an overview of the datasets used in this study. We integrated data of different research groups and from different cell lines.

**Table:** 

Description	Cell line	Stimulus type	Stimulus dose	Reference
recruitment of PARP-1	MEF	microirradiation	405 nm diode laser	Mortusewicz et al.^54^
ATM and MRN recruitment	U2OS, AT	γ-IR, X-ray	0.8–68 Gy	Tobias et al.,^55^ Kozlov et al.^83^
ATM activation	HeLa, HepG2	γ-IR	40 Gy	Stilmann et al.,^26^Hinz et al.^28^
signalosome formation	MEF	γ-IR	80 Gy	Stilmann et al.^26^
TRAF6 complex formation	HeLa, HepG2	γ-IR	30, 40 Gy	Hinz et al.^28^
validation (NF-κB)	HepG2	γ-IR	40 Gy	this study

##### Data for PARP-1 modification

To quantify the recruitment dynamics of PARP-1 to DNA lesions, Mortusewicz et al.^54^ measured the fluorescent intensity of GFP-labeled PARP-1 at the damage site upon inducing DNA damage using microirradiation in single cells. In the study, they measured the intensity of wild type (WT)-PARP-1 for two different time periods with a different temporal resolution (Figure S1, PARP(WT)). To investigate the effect of PARylated PARP-1 on the recruitment dynamics, they quantified the recruitment of an additional PARP-1 variant that is incapable of undergoing PARylation but still binds to the damage site (Figure S1, PARP(E988K)).

##### Data for ATM and MRN recruitment

The recruitment dynamics of MRN and ATM to DNA lesions was investigated and quantified by Tobias et al.^55^ and Kozlov et al.^83^ using time-resolved single cell microscopy with GFP-labeled NBS1, a subunit of the MRN complex, and YFP-labeled ATM (Figure S1, MRN and ATM).

##### Data for ATM activation

Hinz et al.^28^ and Stilmann et al.^26^ measured phosphorylated ATM in the nucleus and cytoplasm upon γ-IR using Western blots (Figure S1, pATM(n) and pATM(c)).

##### Data for signalosome formation

The formation and dissociation of the signalosome was analyzed by Stilmann et al.^26^ using co-immunoprecipitation assays and Western blotting of different components of the signalosome (Figure S1, sig).

##### Data for TRAF6 complex formation

To capture signal propagation in the cytoplasm, we used Western blot data from Hinz et al.^28^ tracking posttranslationally modified TRAF6, TAK1 as well as IKKγ at different time points upon γ-IR (Figure S1, pUbTRAF6, pTAK1 and mUbIKKγ).

##### Data for NF-κB activation (validation)

The activation of NF-κB was monitored using an electrophoretic mobility shift assay (Figure 2B).

#### Mathematical modeling approach

For the development of a quantitative model, we split the signaling pathway of genotoxic IKK/NF-κB activation into smaller modules and created for each module multiple minimal models to investigate different mechanistic hypotheses.^84^ Using mechanistic insights and experimental data from the studies presented in STAR methods, .training and validation data sets enabled us to identify the most appropriate model for each module. By merging the individual models, we created an overall model of genotoxic IKK/NF-κB signaling that is presented in this study.

##### Modeling DNA lesions

To model the recruitment and binding of PARP-1 and MRN to DNA lesions, we implemented a double strand break by defining two binding sites for PARP-1 (BS_P) and two binding sites for MRN (BS_M). Introducing separate binding sites for PARP-1 and MRN prevents an exclusive binding of one of the two components to a binding site and is based on the study of Haince et al. in which PAR molecules, PARP-1 as well as MRE11, a subunit of the MRN complex, colocalize at a break site.^85^ In the experiments that we used to calibrate and validate the model (see STAR methods, training and validation data sets), DNA damage is induced by irradiation of cells for a defined time period. To represent a stimulus-dependent generation of binding sites, we defined the two parameters *kDNA_b* and *DNAb*. While *kDNA_b* determines the rate at which binding sites for PARP-1 and MRN are generated, *DNAb* controls the duration of the stimulus. The parameter *kDNA_b* is calculated by the following formula:

(Equation 10) $$k\_DNAb=r\;\frac{Gy}{s}\cdot 35\frac{DSB}{Gy}\cdot 2\frac{BS}{DSB}$$

In Equation 10, *r* determines the rate of irradiation and is specific for an experiment. Based on the studies of Gulston et al., Loebrich et al. and Prise et al., it is assumed that 1 Gy irradiation causes the generation of 35 DNA double strand breaks.^86^^,^^87^^,^^88^ Moreover, the term is multiplied by a factor of two, to account for the fact that one double strand break leads to two binding sites. For the experimental data capturing the modification of PARP-1 (STAR methods, training and validation data sets), the irradiation dose could not be determined. We therefore fitted the parameter *k_DNAb* for this particular dataset (Table S1). For all other datasets, *k_DNAb* was calculated according to Equation 10. The parameter *DNAb* is set to one for a time period given by the corresponding experiment. After this period, *DNAb* is set to zero. The generation of binding sites is computed by multiplying *kDNA_b* with *DNAb* (see Equation 31).

##### Modeling PARP-1 recruitment to DNA

In the model (see Figure 1A for the reaction scheme, STAR methods for the ODE and reaction rates) we assume that PARP-1 is either directly recruited to the lesion by being attached to the binding site *BS_P* (PARPDSB_b, Equation 32) or indirectly (PARP_b, Equation 34) by the recruitment of already bound and PARylated PARP-1 proteins (parPARP1_b). Our hypothesis is derived from the elaborated study of Mortusewicz et al.^54^ in which the recruitment dynamics of various PARP-1 variants was quantified. We used the recruitment dynamics of WT PARP-1 and a catalytically inactive PARP-1 variant (E988K) to test multiple hypotheses by fitting different variants of minimal models to the data.^84^ The superior model variant was then selected for the overall model. To include the effect of the catalytically inactive PARP-1 variant in the model, we implemented parameter *AB* that is set to one for WT PARP-1 or zero for the inactive E988K variant (Equations 36 and 37).

##### Modeling MRN and ATM recruitment

For the recruitment of the MRN complex to the binding site (BS_M) and the recruitment and activation of ATM, we included positive feedbacks (Equations 40 and 43) to capture the cyclic process of MRN and ATM recruitment to the damage site. Specifically, the phosphorylation of H2AX histones by recruited ATM proteins in proximity of the DNA lesion causes the recruitment of MDC1 and additional MRN complexes. In turn, further ATM molecules are recruited and activated. Thus, MRN and ATM maintain their own recruitment.^89^ We implemented the positive feedbacks by Hill terms that allow reproducing the sigmoidal shaped time course of activated ATM upon DNA damage (Figure S1, pATM(n)). As ATM exists in cells as inactive homodimers or multimers that transform by auto-phosphorylation into active monomers,^48^ we set the Hill coefficient for both Hill terms to a value of two.

##### Modeling IKKγ modification

Upon activation of ATM and PARylation of PARP-1 the signalosome is formed and results in the sumoylation and phosphorylation of IKKγ (Equations 45 and 46). While activated ATM (pATM) mediates the phosphorylation of IKKγ, the SUMO-1 ligase PIASy sumoylates IKKγ. As there was no experimental data available capturing the levels of PIASy over time, we did not include this component in the model.

##### Modeling IKK complex activation

Based on the findings of Hinz et al., phosphorylated ATM as well as modified IKKγ translocate to the cytoplasm where ATM induces the K63-linked polyubiquitination of TRAF6 which in turn facilitates the recruitment and activation of kinase TAK1. The ubiquitin ligase cIAP1 is recruited as well and mediates the mono-ubiquitination of IKKγ. For simplicity and due to lack of sufficient data we neglected cIAP1 in the model. In the model, the translocation of phosphorylated ATM to the cytoplasm is lumped together with the binding and polyubiquitination of TRAF6 (see Equation 47). The binding and activation of TAK1 is modeled in Equation 48. Note that the processes describing the translocation of modified IKKγ into the cytoplasm, mono-ubiquitination, integration into IKK complexes and the activation of the IKK complex by TAK1-mediated phosphorylation of IKKβ are modeled as a single reaction (Equation 49).

##### Ordinary differential equations of the model

Most variable names correspond to the labels in Figure 1. For some variables abbreviated names are introduced.

**Table:** 

name	description
*AMRN_b*	ATM bound to the DNA bound MRN complex
*sig*	signalosome consisting of pATM, parPARP1 and IKKγ
*spIKKγ*	sumoylated and phosphorylated IKKγ
*AT*	complex consisting of pATM and TRAF6
*ATT*	complex consisting of pATM and TRAF6 and pTAK1
*pIKK*	activated IKK complex in which IKKβ is phosphorylated

(Equation 11) $$\frac{d\left(BS\_P\left(t\right)\right)}{dt}=v1-v2+v3$$

(Equation 12) $$\frac{d\left(PARP1\left(t\right)\right)}{dt}=v3+v5+v9+v16-v2-v4$$

(Equation 13) $$\frac{d\left(PARP1DSB\_b\left(t\right)\right)}{dt}=v2-v3-v6$$

(Equation 14) $$\frac{d\left(PARP1\_b\left(t\right)\right)}{dt}=v4-v5-v7$$

(Equation 15) $$\frac{d\left(parPARP1\_b\left(t\right)\right)}{dt}=v6+v7-v8$$

(Equation 16) $$\frac{d\left(parPARP1\left(t\right)\right)}{dt}=v8-v9-v15$$

(Equation 17) $$\frac{d\left(BS\_M\left(t\right)\right)}{dt}=v1-v10-v11$$

(Equation 18) $$\frac{d\left(MRN\left(t\right)\right)}{dt}=-v10-v11$$

(Equation 19) $$\frac{d\left(MRN\_b\left(t\right)\right)}{dt}=v10+v11-v12+v13+v14$$

(Equation 20) $$\frac{d\left(AMRN\_b\left(t\right)\right)}{dt}=v12-v13-v14$$

(Equation 21) $$\frac{d\left(ATM\left(t\right)\right)}{dt}=-v12$$

(Equation 22) $$\frac{d\left(pATM\left(t\right)\right)}{dt}=v13+v14-v15+v16-v17$$

(Equation 23) $$\frac{d\left(IKK\gamma \left(t\right)\right)}{dt}=-v15$$

(Equation 24) $$\frac{d\left(sig\left(t\right)\right)}{dt}=v15-v16$$

(Equation 25) $$\frac{d\left(spIKK\gamma \left(t\right)\right)}{dt}=v16-v19$$

(Equation 26) $$\frac{d\left(TRAF6\left(t\right)\right)}{dt}=-v17$$

(Equation 27) $$\frac{d\left(AT\left(t\right)\right)}{dt}=v17-v18$$

(Equation 28) $$\frac{d\left(ATT\left(t\right)\right)}{dt}=v18$$

(Equation 29) $$\frac{d\left(TAK1\left(t\right)\right)}{dt}=-v18$$

(Equation 30) $$\frac{d\left(pIKK\left(t\right)\right)}{dt}=v19$$

##### Reaction rates of the model

(Equation 31) $$v1=k\_DNAb\cdot DNAb\left(t\right)$$

(Equation 32) $$v2=kon\cdot PARP1\left(t\right)\cdot BS\_P\left(t\right)$$

(Equation 33) $$v3=koff\cdot PARP1DSB\_b\left(t\right)$$

(Equation 34) $$v4=kr\cdot PARP1\left(t\right)\cdot parPARP1\_b\left(t\right)$$

(Equation 35) $$v5=koff2\cdot PARP1\_b\left(t\right)$$

(Equation 36) $$v6=vact\cdot PARP1DSB\_b\left(t\right)\cdot AB$$

(Equation 37) $$v7=vact\cdot PARP1\_b\left(t\right)\cdot AB$$

(Equation 38) $$v8=koff\_act\cdot parPARP1\_b\left(t\right)$$

(Equation 39) v9=kdepar·parPARP1(t)

(Equation 40) $$v10=k1\cdot MRN\left(t\right)\cdot BS\_M\left(t\right)\cdot \frac{MRN\_b^{n}\left(t\right)}{km+MRN\_b^{n}\left(t\right)}$$

(Equation 41) $$v11=k4\cdot MRN\left(t\right)\cdot BS\_M\left(t\right)$$

(Equation 42) $$v12=k2\cdot ATM\left(t\right)\cdot MRN\_b\left(t\right)$$

(Equation 43) $$v13=k3\cdot AMRN\_b\left(t\right)\cdot \frac{pATM^{n}\left(t\right)}{km2+pATM^{n}\left(t\right)}$$

(Equation 44) $$v14=k5\cdot AMRN\_b\left(t\right)$$

(Equation 45) $$v15=SFM\_k1\cdot parPARP1\left(t\right)\cdot pATM\left(t\right)\cdot IKK\gamma \left(t\right)$$

(Equation 46) $$v16=SFM\_k2\cdot sig\left(t\right)$$

(Equation 47) $$v17=TM\_k1\cdot pATM\left(t\right)\cdot TRAF6\left(t\right)$$

(Equation 48) $$v18=TM\_k2\cdot AT\left(t\right)\cdot TAK1\left(t\right)$$

(Equation 49) $$v19=TM\_k3\cdot spIKK\gamma \left(t\right)\cdot ATT\left(t\right)$$

##### Conserved moieties in the model

(Equation 50) PARP1_tot=PARP1+PARP1DSB_b+PARP1_b+parPARP1_b+parPARP1+sig

(Equation 51) MRN_tot=MRN+MRN_b+AMRN_b

(Equation 52) ATM_tot=ATM+AMRN_b+pATM+sig+AT+ATT

(Equation 53) IKKγ_tot=IKKγ+sig+spIKKγ+pIKK

(Equation 54) TRAF6_tot=TRAF6+AT+ATT

(Equation 55) TAK1_tot=TAK1+ATT

##### Defining activated IKK complex as readout

For our analyses, we defined the activated IKK complex as a readout for NF-κB activity. The IKK complex is a direct activator of NF-κB as it phosphorylates and thereby initiates the degradation of IκBs which inhibit NF-κB. In addition, the IKK complex phosphorylates RelA/p65. Upon degradation of IκBs, NF-κB translocates into the nucleus and induces the expression of its target genes. Since we here aimed to model the initial activating processes that are specific for the genotoxic pathway we focused on the upstream processes leading to genotoxic stress-induced NF-κB activation. As NF-κB dynamics closely follow the dynamics of activated IKK (Figure 2B) we selected the activated IKK complex as the readout of our model.

##### Model simulations and parameter inference

For simulations and parameter inference we used MATLAB (R2017b, The Mathworks Inc., Natick, MA) in combination with the open source toolbox Data2Dynamics.^79^^,^^80^ For parameter inference, we set the bounds of parameters to −10 and 3 on a log_10_ scale if not stated otherwise (Table S1). Variables and parameters in the model have units based on number of molecules (m) and seconds (s). For some of the parameters the boundaries were adapted to include information about experimentally quantified reaction rates from literature or to prevent numerical issues during simulation. For the half-life of PARylated PARP-1 (parPARP1) a time range of one to 6 min was published^70^ which corresponds to an exponential decay rate of −1.9 and −2.6 s^−1^ on a log_10_ scale. We therefore set the boundaries of the corresponding parameter *kdepar* to −4 and 0 s^−1^ (Table S1). For the PARylation of PARP-1 a rate of 0.41 s^−1^ and 5 s^−1^ was reported.^68^^,^^69^ The total concentrations of the conserved moieties ATM, IKK, MRN, TAK1 and TRAF6 were fitted and the boundaries were set to 2 and 7 on a log_10_ scale to ensure that the estimated values are in a physiological range. The total amount of PARP-1 was set to 1.9x10^5^ molecules in accordance to Schwanhäusser et al.^64^

The optimization is based on maximum likelihood estimation and the MATLAB in-built function lsqnonlin was used for minimizing the objective function. To prevent local minima, multi-start optimization was used. The parameter values of the best fit are given in Table S1.

To assess the identifiability of fitted parameters, we used a profile likelihood-based approach^90^ and applied it to each kinetic parameter of the model (Figure S3). The analysis shows that almost all estimated parameters are identifiable, i.e., there exists a finite range for the confidence interval of a parameter. In contrast, for the parameters MRN_tot, TAK1_tot, TRAF6_tot, kdepar and koff2 only the lower or upper limit of the confidence interval can be properly determined as the upper or lower boundary of the parameter value is reached. For example, for MRN_tot the lower limit of the confidence interval can be determined, but for the upper limit, the parameter boundary is reached at 10^7^ molecules. Overall, the results show that the majority of parameters is identifiable with the included datasets.

##### Link between modified IKKγ and IKK complex

Based on the model structure and the implementation of processes, it is possible to comprehend that the amount of modified IKKγ (spIKKγ) produced over time corresponds to the amount of activated IKK complex in the stimulated steady state. This is due to i) the assumed irreversible translocation and integration of modified IKKγ into IKK complexes (parameter TM_k3 in Figures 1A and Equation 49) and ii) the implementation of IKKγ as a conserved moiety and iii) a catalytic function of the cytoplasmic TRAF6 complex (ATT) for the activation of IKK (parameter TM_k3 in Figures 1A and Equation 49), i.e., there is no mass flow from the TRAF6 complex to the IKK complex. As the cytoplasmic TRAF6 complex is not degraded and does not dissociate in the model, it cannot restrict the amount of modified IKKγ that is integrated into the IKK complex once the TRAF6 complex is formed.

##### Sensitivity analysis

To identify processes with the strongest impact on inhibiting IKK/NF-κB activity under genotoxic stress, we performed a sensitivity analysis for all kinetic model parameters by reducing each parameter individually by 90%. In order to investigate the sensitivity of ATM activation, we moreover reduced the two parameters k3 and k5 simultaneously by 90%. The activated IKK complex (pIKK) in stimulated steady state was chosen as readout for the analysis. The sensitivity coefficients (sc) for the specified parameters were calculated for various irradiation doses ranging from 1 Gy to 100 Gy by using the following formula:

(Equation 56) $$sc\left(p,i\right)=\frac{{\bar{pIKK\left(p,i\right)}}^{*}-\bar{pIKK\left(i\right)}}{\bar{pIKK\left(i\right)}}$$

${\bar{pIKK}}^{*}$ represents the level of activated IKK complex in the stimulated steady state for irradiation dose *i* upon the perturbation of a parameter *p*. $\bar{pIKK}$ represents the level of activated IKK complex in the stimulated steady state without perturbation of parameter *p*. The most effective inhibition of IKK complex activation is characterised by parameter perturbations causing a reduction in the level $\bar{pIKK}$ to a value of zero which results in a sensitivity coefficient of −1, given $\bar{pIKK}$ is nonzero. Parameter perturbations causing an increase in IKK complex activation can result in sensitivity coefficients higher than +1 as there is no upper limit for positive coefficients.

### Quantification and statistical analysis

For the quantification of Western blots and electrophoretic mobility shift assay data, we used the software ImageJ.^78^ To determine the intensity of protein bands, the ImageJ in-built functions for polyacrylamide gel analysis was applied. The band intensity of the protein of interest was normalized to the corresponding protein band of the loading control for the Western blots.

## Data Availability

• All original model code has been deposited at the BioModels database^81^ and is publicly available at https://www.ebi.ac.uk/biomodels/MODEL2307130001. The model identifier is in the key resources table. • All data reported in this paper will be shared by the lead contact upon request. • Any additional information required to reanalyze the data reported in this paper is available from the lead contact upon request.
