# Supplementary material for: A computational model of the DNA damage-induced IKK/ NF-κB pathway reveals a critical dependence on irradiation dose and PARP-1
Source: iScience. 2023 Sep 14;26(10):107917. doi: 10.1016/j.isci.2023.107917 (PMC10561052; doi:10.1016/j.isci.2023.107917)
Supplement: Document S1. Figures S1–S3 and Table S1 [file mmc1.pdf]

iScience, Volume *26*

Supplemental information

A computational model of the DNA damage-induced IKK/ NF-κB pathway reveals a critical dependence on irradiation dose and PARP-1

Fabian Konrath, Michael Willenbrock, Dorothea Busse, Claus Scheidereit, and Jana Wolf

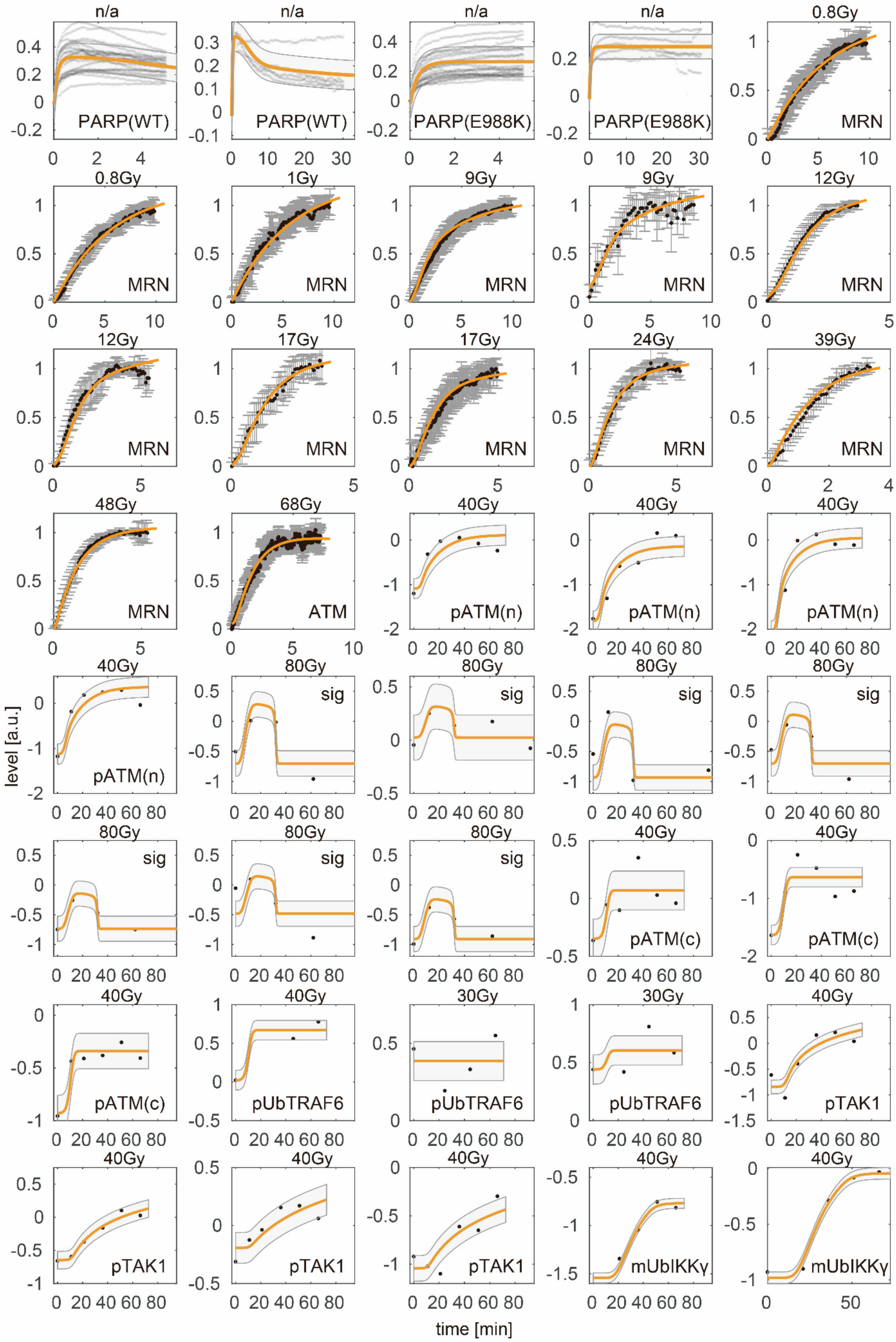

n/a
PARP(WT)
n/a
PARP(WT)
n/a
PARP(E988K)
n/a
PARP(E988K)
0.8Gy
MRN
0.8Gy
MRN
1Gy
MRN
9Gy
MRN
9Gy
MRN
12Gy
MRN
12Gy
MRN
17Gy
MRN
17Gy
MRN
24Gy
MRN
39Gy
MRN
48Gy
MRN
68Gy
ATM
40Gy
pATM(n)
40Gy
pATM(n)
40Gy
pATM(n)
40Gy
pATM(n)
80Gy
sig
80Gy
sig
80Gy
sig
80Gy
sig
80Gy
sig
80Gy
sig
80Gy
sig
40Gy
pATM(c)
40Gy
pATM(c)
40Gy
pATM(c)
40Gy
pUbTRAF6
30Gy
pUbTRAF6
30Gy
pUbTRAF6
40Gy
pTAK1
40Gy
pTAK1
40Gy
pTAK1
40Gy
pTAK1
40Gy
mUbIKKγ
40Gy
mUbIKKγ
level [a.u.]
time [min]

Fig S1. Experimental data and simulations of calibrated model, related to Figure 1 and STAR Methods.

Dots represent experimental data and the yellow line represents simulation data. The grey area surrounding the yellow line shows the fitted standard deviation. Otherwise the standard deviation is represented by grey bars which is true for the MRN and ATM recruitment data. For the recruitment data of the PARP-1 WT and mutant two similar data sets exist which differ in the temporal resolution and time period. For those experiments, the irradiation dose is not available (n/a). For 0.8, 12, and 17 Gy for the recruitment data of the MRN complex, additional biological replicates exist. Details about the experimental data and settings are given in STAR methods. The estimated parameter values for the best fit are shown in Table S1. Abbreviation: sig: signalosome.

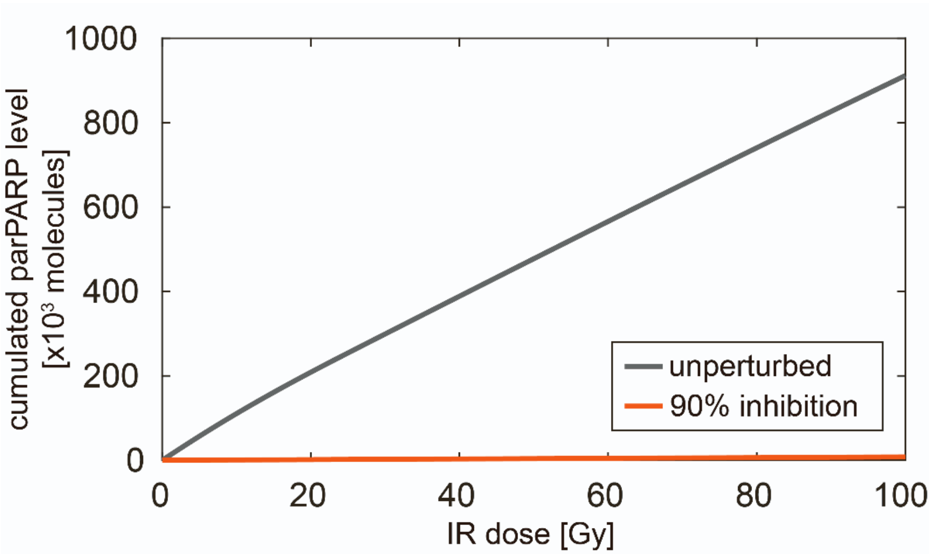

Fig S2. Influence of irradiation dose and PARP-1 inhibition on cumulated amount of parPARP, related to Figure 4 and Figure 5.

The cumulated amount of parPARP was quantified by integrating the flux of PARylated PARP-1 dissociating from DNA (corresponding parameter: koff_act) in the time frame of signalosome formation for various irradiation doses with and without 90% inhibition of PARylation (corresponding parameter: vact).

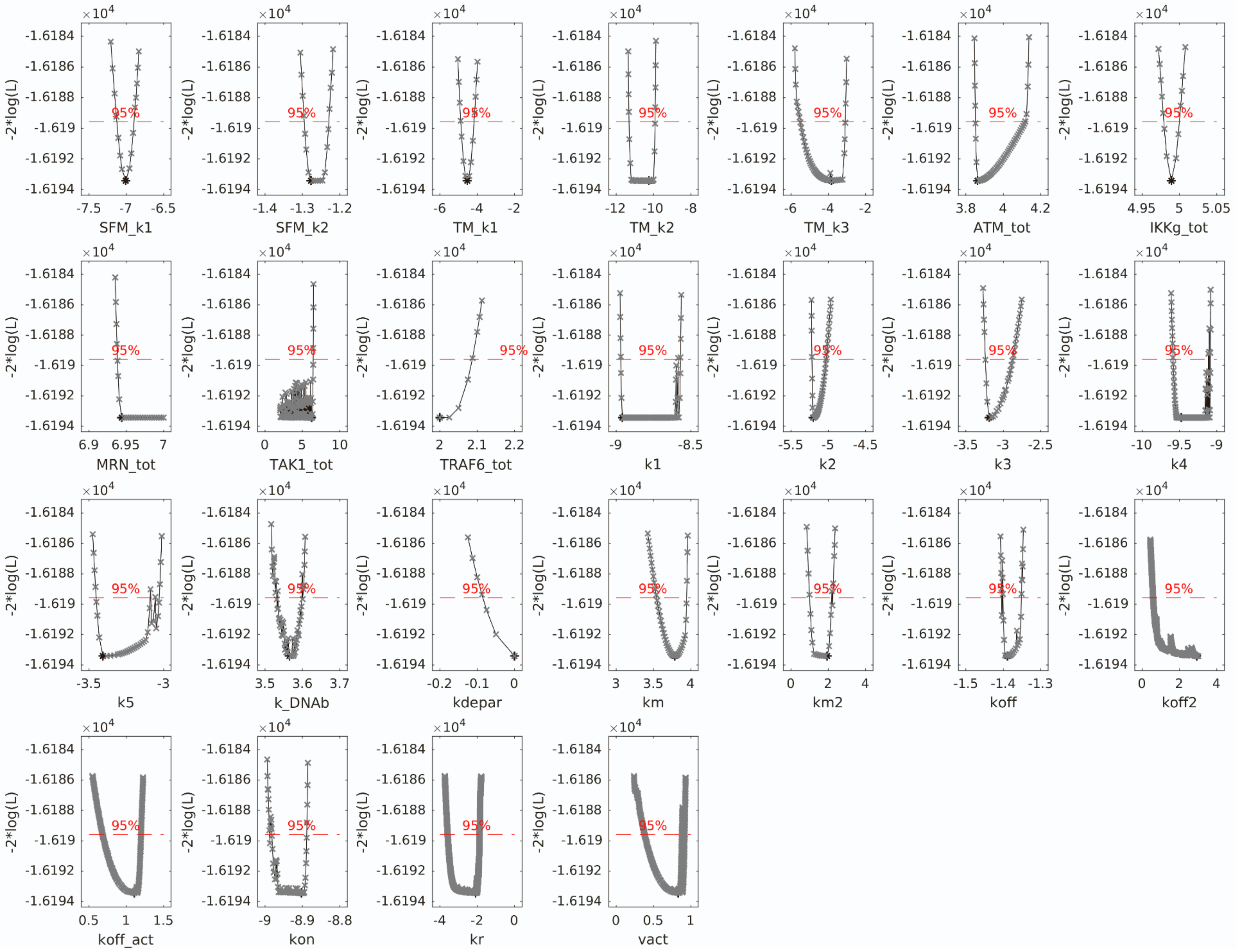

×10^4
-2*log(L)
95%
-1.6184
-1.6186
-1.6188
-1.619
-1.6192
-1.6194
SFM_k1
SFM_k2
TM_k1
TM_k2
TM_k3
ATM_tot
IKKg_tot
MRN_tot
TAK1_tot
TRAF6_tot
k1
k2
k3
k4
k5
k_DNAb
kdepar
km
km2
koff
koff2
koff_act
kon
kr
vact

Fig S3. Profile likelihoods for estimated model parameters, related to STAR Methods.

The black star represents the estimated parameter value of the best fit and the corresponding fit quality given as negative log likelihood. To compute the profile likelihood, the specified parameter is fixed to an increased or decreased value compared to the calibrated value and the remaining parameters are refitted. The resulting log likelihood is represented by a grey x. To identify the 95% confidence interval of a parameter, a threshold for the log likelihood is calculated based on a χ^2 distribution with one degree of freedom (dashed red line).

Table S1. Parameter description with estimated parameter values, related to STAR methods.

label	log_{10} value		log_{10} values of boundaries	description
SFM_k1	-7.0	$m^{-2}\cdot s^{-1}$	[-10, -2]	formation of signalosome
SFM_k2	-1.3	s^{-1}	[-10, 3]	dissociation of signalosome
TM_k1	-4.5	$m^{-1}\cdot s^{-1}$	[-10, 3]	poly-ubiquitination of TRAF6 and binding of pATM
TM_k2	-10.2	$m^{-1}\cdot s^{-1}$	[-12, 3]	activation of TAK1 and recruitment to TRAF6 complex
TM_k3	-3.8	$m^{-1}\cdot s^{-1}$	[-10, 3]	integration of IKKγ into IKK complex, mono-ubiquitination of IKKγ and phosphorylation of IKKβ
ATM_tot	3.9	m	[2, 7]	total concentration of ATM
IKKγ_tot	5.0	m	[2, 7]	total concentration of IKKγ
MRN_tot	6.9	m	[2, 7]	total concentration of MRN
PARP1_tot	5.3	m	[-, -]	total concentration of PARP-1
TAK1_tot	6.2	m	[2, 7]	total concentration of TAK1
TRAF6_tot	2.0	m	[2, 7]	total concentration of TRAF6
k1	-9.0	$m^{-1}\cdot s^{-1}$	[-10, 3]	positive feedback of chromatin-bound MRN on MRN recruitment
k2	-5.2	$m^{-1}\cdot s^{-1}$	[-10, 3]	recruitment of ATM to MRN
k3	-3.2	s^{-1}	[-10, 1]	positive feedback of pATM on activation of ATM
k4	-9.5	$m^{-1}\cdot s^{-1}$	[-10, 3]	recruitment of MRN to DNA lesion
k5	-3.4	s^{-1}	[-10, 1]	activation of ATM and its dissociation from MRN
k_DNAb	3.6	$m\cdot s^{-1}$	[2, 4]	generation of binding sites
kdepar	0.0	s^{-1}	[-4, 0]	dePARylation of PARP-1
km	3.8	m^2	[-4, 9]	parameter of Hill term for positive feedback of MRN
km2	1.9	m^2	[-4, 9]	parameter of Hill term for positive feedback of ATM
koff	-1.4	s^{-1}	[-10, 3]	dissociation of PARP-1 from DNA lesion
koff2	2.9	s^{-1}	[-10, 3]	dissociation of PARP-1 from periphery of lesion
koff_act	1.1	s^{-1}	[-10, 3]	dissociation of PARylated PARP-1 from chromatin
kon	-8.9	$m^{-1}\cdot s^{-1}$	[-9, -2]	binding of PARP-1 to lesion

kr	-2.1 $m^{-1} \cdot s^{-1}$	[-9, 3]	PARylated PARP-1-induced recruitment of PARP-1 to periphery of lesions
n	$\log_{10}(2)$	[-, -]	Hill coefficient for positive feedback of MRN and ATM
vact	0.8 s^{-1}	[-0.4, 1]	automodification of PARP-1

Abbreviations: m: molecules, s: seconds
